# Distinct regional allergen sensitization patterns in pediatric populations: A comparative latent class analysis of multiple antigen simultaneous test-derived sensitization profiles in Japan and Taiwan

**DOI:** 10.5415/apallergy.0000000000000223

**Published:** 2025-12-02

**Authors:** Ching-Wei Lin, Su-Boon Yong, Rei Kanai, Lawrence Shih-Hsin Wu, Pei-Chi Chen, Xiao-Ling Liu, Hui-Fang Kao, Mizuho Nagao, Yasunori Sato, Chang-Ching Wei, Jiu-Yao Wang, Takao Fujisawa

**Affiliations:** 1 Department of Pediatrics, National Cheng Kung University Hospital, College of Medicine, National Cheng Kung University, Tainan, Taiwan; 2 Department of Allergy and Immunology, China Medical University Children’s Hospital, China Medical University, Taichung, Taiwan; 3 Allergy Center and Department of Clinical Research, NHO Mie National Hospital, Tsu, Japan; 4 Graduate Institute of Biomedical Sciences, China Medical University, Taichung, Taiwan; 5 Department of Microbiology and Immunology, College of Medicine, National Cheng Kung University, Tainan, Taiwan; 6 Research Center of Allergy, Immunology and Microbiome (A.I.M.), China Medical University Hospital, China Medical University, Taichung, Taiwan; 7 Department of Nursing, National Tainan Junior College of Nursing, Tainan, Taiwan; 8 Department of Biostatistics, Keio University Graduate School of Medicine, Tokyo, Japan

**Keywords:** Allergen profiling, cross-national comparison, latent class analysis, MAST, pediatric allergy

## Abstract

**Background:**

Allergen sensitization patterns vary across regions; however, few studies have directly compared pediatric sensitization profiles between Japan and Taiwan.

**Objective:**

This study aimed to compare allergen sensitization patterns in children from Japan and Taiwan using the multiple antigen simultaneous test (MAST) and latent class analysis (LCA).

**Methods:**

A total of 217 Japanese and 166 Taiwanese children (aged 2–12 years) were enrolled. Based on responses to the International Study on Asthma and Allergy in Childhood questionnaire, participants were classified as either healthy or as having allergic rhinitis (AR), bronchial asthma (BA), or atopic dermatitis. Serum samples were analyzed using a commercial MAST kit available in Japan, and 20 allergens shared with the panel used in Taiwan were selected for analysis. Sensitization was defined as a lumicount value ≥2.78 (equivalent to class 2 or higher). LCA was conducted on the combined dataset, and the optimal number of latent classes was determined using the Akaike Information Criterion.

**Results:**

LCA identified 4 latent classes: low-sensitized (LS), house dust mite–dominant (HDM-D), inhalant-sensitized (IH-S), and poly-sensitized (PS). The HDM-D class was more prevalent in Taiwan (50%) than in Japan (6.9%). In contrast, the IH-S and PS classes were more common in Japan (36.4% vs. 3% and 8.8% vs. 2.4%, respectively). AR and BA were primarily associated with HDM-D in Taiwan and with IH-S in Japan, which includes HDM and Japanese cedar pollen. HDM and crab sensitization were strongly correlated in Taiwan.

**Conclusion:**

MAST combined with LCA reveals distinct regional sensitization profiles, suggesting divergent pathways in allergic disease development.

## Introduction

Allergenic sources vary widely, ranging from complex biological mixtures such as pollen and house dust mites (HDMs) to well-defined chemical molecules found in occupational agents and drugs. In Asia, differences in environmental and dietary exposures have contributed to distinct regional allergen profiles. For instance, in Taiwan, HDMs and food allergens—specifically crab, clam, shrimp, peanut, and beef—are frequently implicated in allergic reactions [1–6]. In contrast, allergen profiles in Japan often feature sensitization to pollen from cedar and cypress trees, as well as to HDMs and dietary allergens such as shellfish, egg, and milk [7, 8].

Epidemiological evidence supports these regional variations. A Taiwanese study conducted between 2018 and 2022 reported that HDMs, particularly *Dermatophagoides farinae* (41.8%) and *Dermatophagoides pteronyssinus* (37.9%), were among the most prevalent allergens. Food allergens such as crab (12.6%), clam (9.8%), shrimp (9.1%), peanut (7.8%), and beef also played significant roles [9]. The prevalence of food allergy in Taiwanese children increased from 7.7% to 10.4% between 2004 and 2017, with shrimp and crab being particularly common, and peanut allergy rising to 1.1% [4]. In Japan, a birth cohort study showed that the major allergen components at age 9 were Der f 1 (HDM) and Cry j 1 (Japanese cedar), with prevalence rates of 74.8% and 57.8%, respectively [7]. Sensitization to PR-10 cross-reactive components, such as Bet v 1 (birch) and Pru p 1 (peach), was also common [7]. Changing environmental conditions may influence sensitization patterns [10].

The multiple antigen simultaneous test (MAST) enables the simultaneous detection of specific immunoglobulin E (IgE) antibodies to a broad panel of allergens. While it lacks the molecular resolution of component-resolved diagnostics, its multiplex nature provides a population-level overview of sensitization patterns, offering valuable insight into the allergenic landscape and its clinical implications [1].

Despite these insights, few studies have directly compared allergen sensitization profiles in pediatric populations across East Asian countries using standardized testing platforms and data-driven classification methods. To address this gap, the present study aimed to identify and compare latent allergen sensitization patterns in children from Japan and Taiwan using regionally adapted MAST panels in conjunction with latent class analysis (LCA), with a focus on exploring potential regional differences in sensitization profiles and their associations with allergic diseases.

## Materials and methods

### Study participants

Children aged 2 to 12 years were recruited from the general pediatric population as well as from those clinically diagnosed with asthma and receiving treatment at affiliated clinics. Recruitment was conducted using multiple approaches including our hospital’s website, collaboration with local schools, and posters displayed at National Cheng Kung University Hospital in Tainan, Taiwan, and National Hospital Organization Mie Hospital in Mie, Japan. Before enrollment, participants and their guardians received detailed written information regarding the study’s purpose and methods, and written informed consent was obtained.

### Clinical assessments

All participants completed the International Study on Asthma and Allergy in Childhood (ISAAC) questionnaire, which provided systematic documentation of physician-diagnosed allergic diseases, such as asthma, perennial allergic rhinitis (AR), seasonal AR, hay fever, atopic dermatitis (AD), and food allergies, as well as information regarding dietary exclusions [11].

### Multiple antigen simultaneous test procedure

Serum samples from all participants were analyzed for allergen sensitization using the MAST, commercially available in Japan. This assay detects allergen-specific IgE via a chemiluminescent reaction, and the resulting signal (“lumicount”) is quantified. In this study, we analyzed both the presence of sensitization—defined as a lumicount value ≥2.78 (corresponding to class 2 or higher)—and the distribution of lumicount values [1]. Although the MAST panels available in Japan and Taiwan each include up to 36 allergens, we limited our analysis to 20 allergens common to both. This approach was adopted to ensure the comparability of results between countries and to enhance the real-world applicability of our findings in clinical settings where only shared testing items can be evaluated. These allergens included inhalant types—such as house dust, *D. farinae*, dog dander, cat dander, Alternaria, Aspergillus, Japanese cedar pollen, timothy grass pollen, and a ragweed mix (*Ambrosia artemisiifolia* and *Ambrosia trifida*)—as well as food and other allergens, including latex, egg white, milk, peanut, soybean, wheat, shrimp, crab, pork, beef, and tuna.

### Statistical analysis

LCA was applied to the MAST data to identify qualitatively distinct sensitization profiles among the enrolled children based on their responses to the 20 common allergens. LCA is based on the premise that unobserved subgroups within a heterogeneous population can be inferred from observed patterns across various assessment indicators [12, 13]. LCA assumes that the heterogeneity in observed IgE response patterns is attributable to membership in unobserved latent subgroups. Model fit and the optimal number of classes were determined using log-likelihood, the Bayesian Information Criterion (BIC) [14, 15] and the Akaike Information Criterion (AIC) [16]. Given the exploratory nature of this study and the potential risk of underestimating the number of latent classes, we prioritized model selection based on AIC, which tends to favor more complex models. In addition to statistical indices, model interpretability was also considered in the selection process. LCA and model selection were conducted for the entire datasets using JMP® version 18.

Since lumicount values obtained from MAST were not normally distributed, continuous variables were compared between groups using the Mann-Whitney U test. Categorical variables were analyzed using the chi-square test or Fisher’s exact test, as appropriate. The chi-square test was applied when all expected cell counts were ≥5, whereas Fisher’s exact test was used for smaller sample sizes or sparse data where chi-square assumptions were not met. All statistical analyses and data visualization were performed using GraphPad Prism® version 10. A Venn diagram was generated with the assistance of ChatGPT-supported data processing and visualization.

### Ethics statement

This study protocol was approved by the Ethics Review Committee of the National Hospital Organization Mie Hospital (approval no. 24-25) and by the Ethics Committee of National Cheng Kung University (approval no. B-ER-109-280).

## Results

### Demographic and clinical characteristics

A total of 217 children from Japan and 166 children from Taiwan were enrolled, comprising both healthy subjects and those with allergic diseases (Table 1). The average age was similar between the cohorts (Japan: 7.3 ± 2.9 years; Taiwan: 7.2 ± 2.9 years; no significant difference). However, the proportion of male participants was significantly higher in the Taiwan group (69.6%) compared with the Japan group (52.5%).

**Table 1.** Demographic and clinical features of enrolled subjects

	Japan (n = 217)	Taiwan (n = 166)	*P*-value
Age, mean ± SD	7.3 ± 2.9	7.1 ± 2.8	0.498*
Range	2–12	2–12	
Gender†			
Male	114 (52.5)	103 (69.6)	0.001‡
Female	103 (47.5)	45 (30.4)	
Status of allergic diseases			
Healthy (no allergic disease)	64 (29.5)	46 (27.7%)	<0.0001§
Atopic dermatitis	58 (26.7)	11 (6.6%)	
Asthma	54 (24.9)	60 (36.1%)	
Allergic rhinitis	125 (57.6)	90 (54.2%)	

* Unpaired t test.

† Gender status data missing for 18 individuals.

‡ Chi-square test.

§ Fisher’s exact test.

The prevalence of allergic diseases significantly differed between the Japanese and Taiwanese cohorts. AD was substantially more common among Japanese children, whereas bronchial asthma (BA) was more frequently observed in Taiwanese children. The prevalence of AR was similarly high in both countries, at 57.6% in Japan and 54.2% in Taiwan. Notably, no participants reported having food allergies in the questionnaire responses.

**Figure 1.**
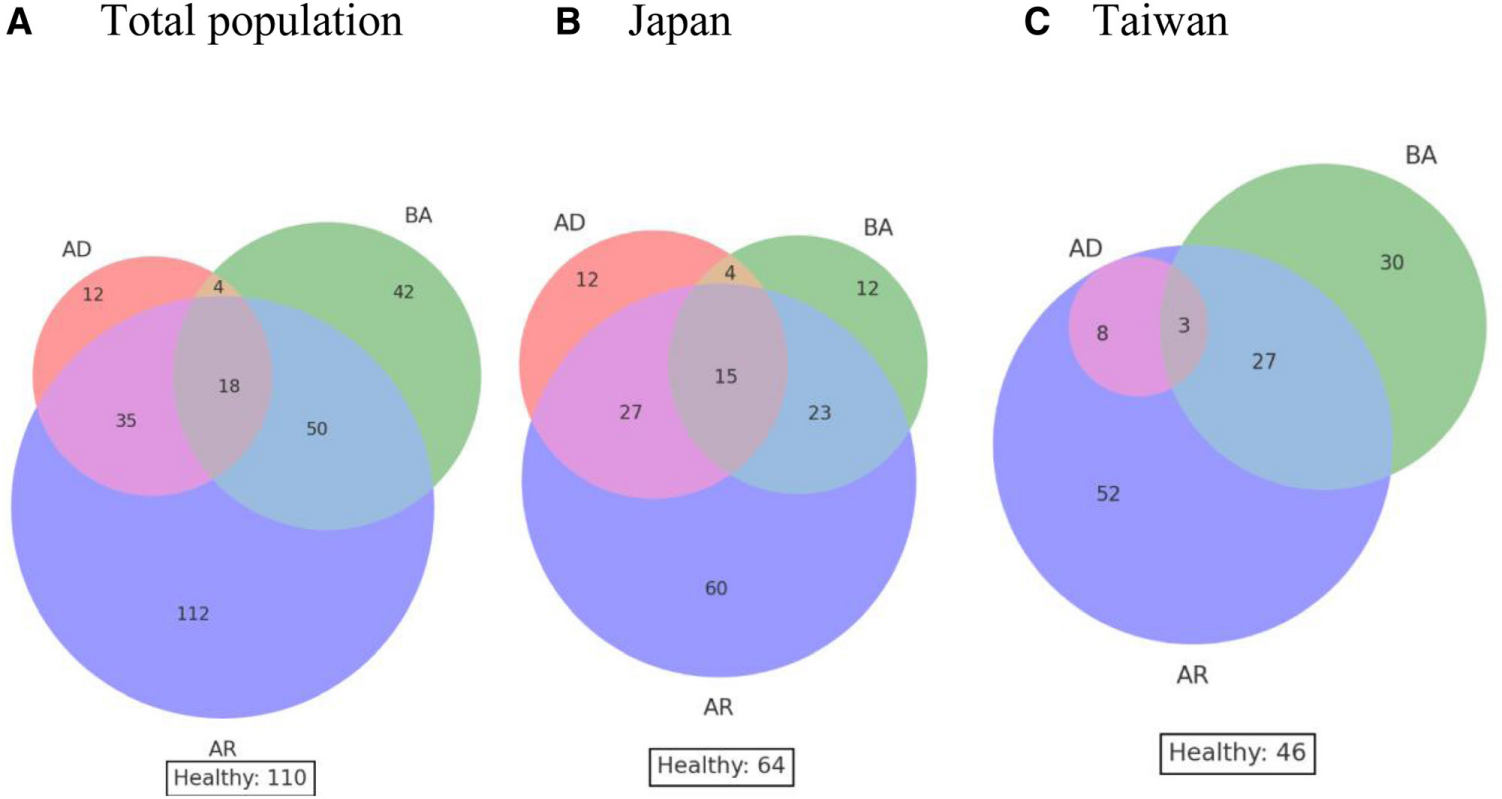
Venn diagram of comorbid allergic diseases in the study population. (A) Total population, (B) Japanese children, (C) Taiwanese children. The overlap of allergic rhinitis, asthma, and atopic dermatitis is illustrated for each group.

Figure 1 illustrates the comorbidity of AD, BA, and AR in the overall sample (a), as well as separately for Japan (b) and Taiwan (c). In both populations, a considerable proportion of children exhibited overlapping allergic conditions. However, isolated BA was more commonly observed in Taiwan, whereas isolated AD was found exclusively in Japan.

**Figure 2.**
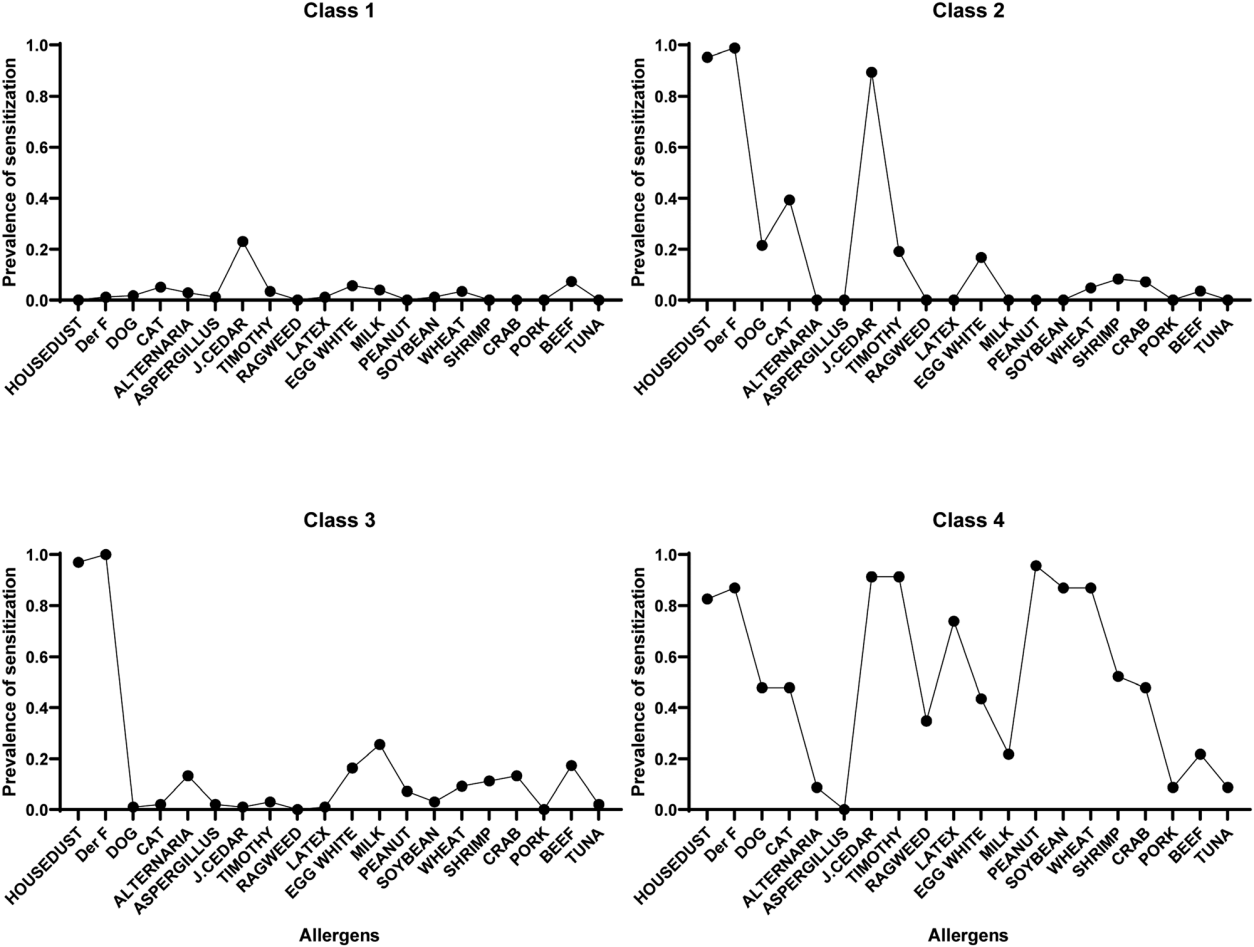
Four sensitization patterns identified through latent class analysis (LCA). The 383 participants were classified into 4 latent classes based on their sensitization profiles to 20 allergens. Each class represents a distinct pattern of allergen-specific immunoglobulin E (IgE) reactivity.

We performed latent class analysis (LCA) to identify sensitization patterns using specific IgE data from 20 allergens. The model with four latent classes showed the best fit according to the BIC value. Figure 2 illustrates the four latent classes derived from the sensitization profiles, highlighting the distinct patterns of allergen-specific IgE reactivity among the 383 participants.

### Latent class analysis using a common allergen panel

Table 2 summarizes the model fit statistics for the 3-class and 4-class solutions. The 3-class model yielded the lowest BIC (3961.78), while the 4-class model showed a better log-likelihood (−1740.82) and the lowest AIC (3647.65). Although BIC slightly favored the 3-class model, the 4-class model was selected as the final model based on its lower AIC, consistent with the exploratory aims of this study.

**Table 2.** Model fit statistics for latent class models

Number of classes	Log-likelihood	BIC	AIC	Selected criterion
3	−1796.5	3961.78	3717	Minimum BIC
4	−1740.82	3975.33	3647.65	Minimum AIC (selected)

AIC, Akaike Information Criterion; BIC, Bayesian Information Criterion.

**Figure 3.**
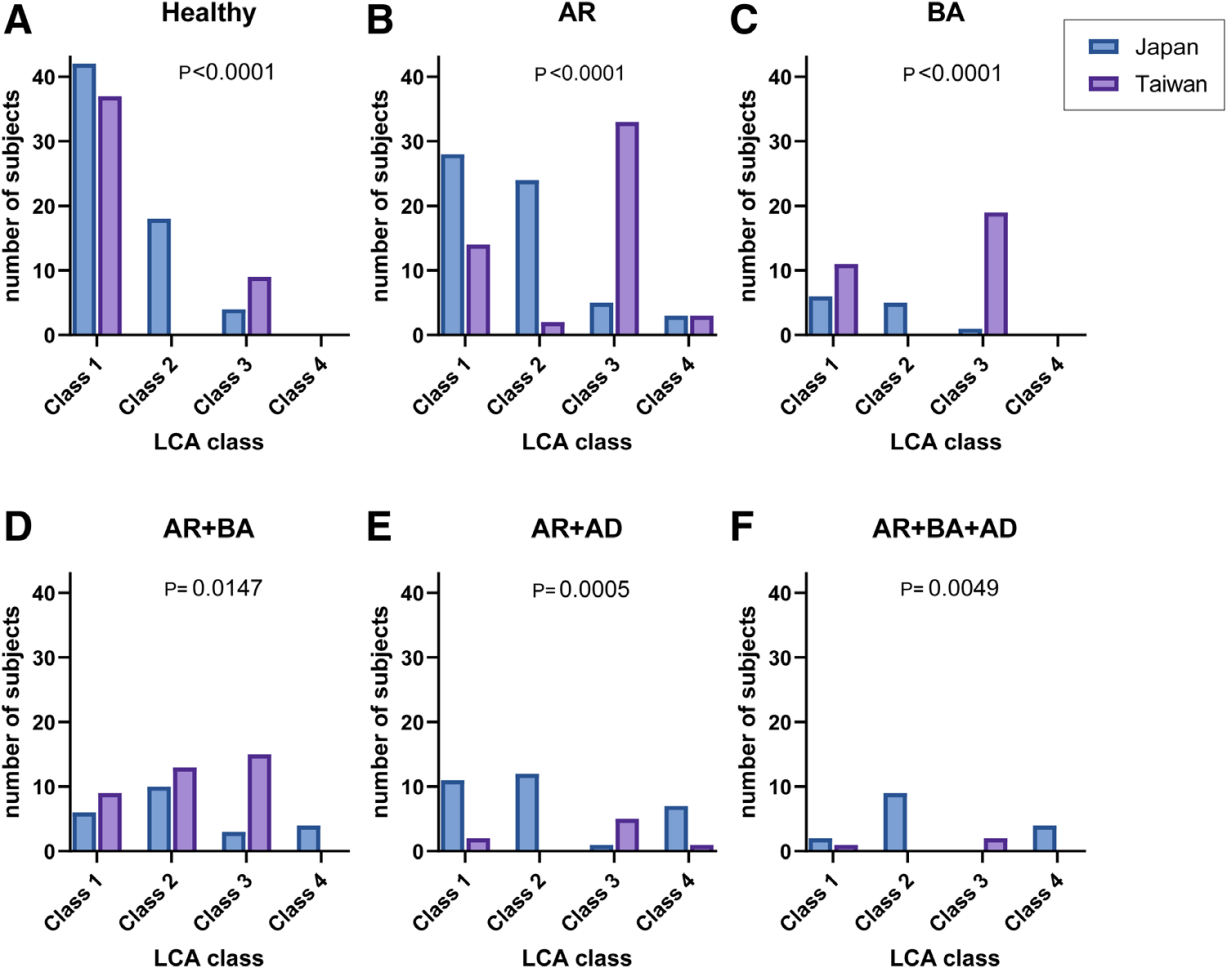
Differences in class distributions in allergic disease status between Japan and Taiwan. The proportions of participants with allergic rhinitis, asthma, and atopic dermatitis are shown across the 4 latent classes in each country.

The selected 4-class model also demonstrated acceptable interpretability, with each class showing distinct and clinically meaningful allergen sensitization patterns. Based on these profiles (Fig. 3), the 4 classes were characterized and labeled as follows:

Class 1: Exhibited generally low sensitization across all tested allergens and was designated the low-sensitized (LS) class.

Class 2: Characterized by sensitization to inhalant allergens, including house dust, dog, cat, and pollens such as Japanese cedar, and labeled the inhalant-sensitized (IH-S) class.

Class 3: Displayed predominant sensitization to HDMs and was defined as the house dust mite–dominant (HDM-D) class.

Class 4: Demonstrated broad sensitization to multiple allergen types and was named the poly-sensitized (PS) class.

### Proportion of sensitized individuals and allergic diseases distribution by latent class

The distributions of latent classes identified by LCA differed significantly between Japan and Taiwan. In Japan, the largest proportion of individuals belonged to class 1 (47.9%), followed by class 2 (36.4%). In contrast, in Taiwan, 50% of participants were assigned to class 3 and 44.6% to class 1, while classes 2 and 4 accounted for only small proportions (Table 3).

**Table 3.** Proportion of sensitized individuals in each LCA class

LCA class	Japan	Taiwan
	N (%)	N (%)
Class 1 (LS)	104 (47.9)	74 (44.6)
Class 2 (IH-S)	79 (36.4)	5 (3.0)
Class 3 (HDM-D)	15 (6.9)	83 (50.0)
Class 4 (PS)	19 (8.8)	4 (2.4)

Fisher’s exact test *P* < 0.0001.

HDM-D, house dust mite–dominant; IH-S, inhalant-sensitized; LCA, latent class analysis; LS, low-sensitized; PS, poly-sensitized.

To further characterize the LCA classes, we examined class distributions across different allergic statuses in both countries (Fig. 3). Among healthy participants, class 1 (LS) was the most prevalent in both cohorts, as expected. Notably, class 2 (IH-S) was the second most common in Japan, whereas class 3 (HDM-dominant) followed in Taiwan (Fig. 3A).

For participants with isolated AR, class 3 was dominant in Taiwan, while classes 1 and 2 were predominant in Japan (Fig. 3B). A similar pattern was observed for isolated BA, where class 3 was most frequent in Taiwan (Fig. 3C).

In cases of comorbid AR and BA, class 3 was again the most frequent in Taiwan, whereas class 2 was predominant in Japan (Fig. 3D). For less common comorbidity patterns, such as AR + AD and AR + BA + AD, class distributions also differed significantly between the 2 countries, once again demonstrating a higher contribution of class 3 in Taiwan and classes 2 and 4 in Japan (Fig. 3E, F).

### Differences in lumicount values (allergen-specific IgE levels) by class between Japan and Taiwan

To better quantify the allergen sensitization profiles in both countries, representative specific IgE lumicount values were compared between Japan and Taiwan for each latent class.

**Figure 4.**
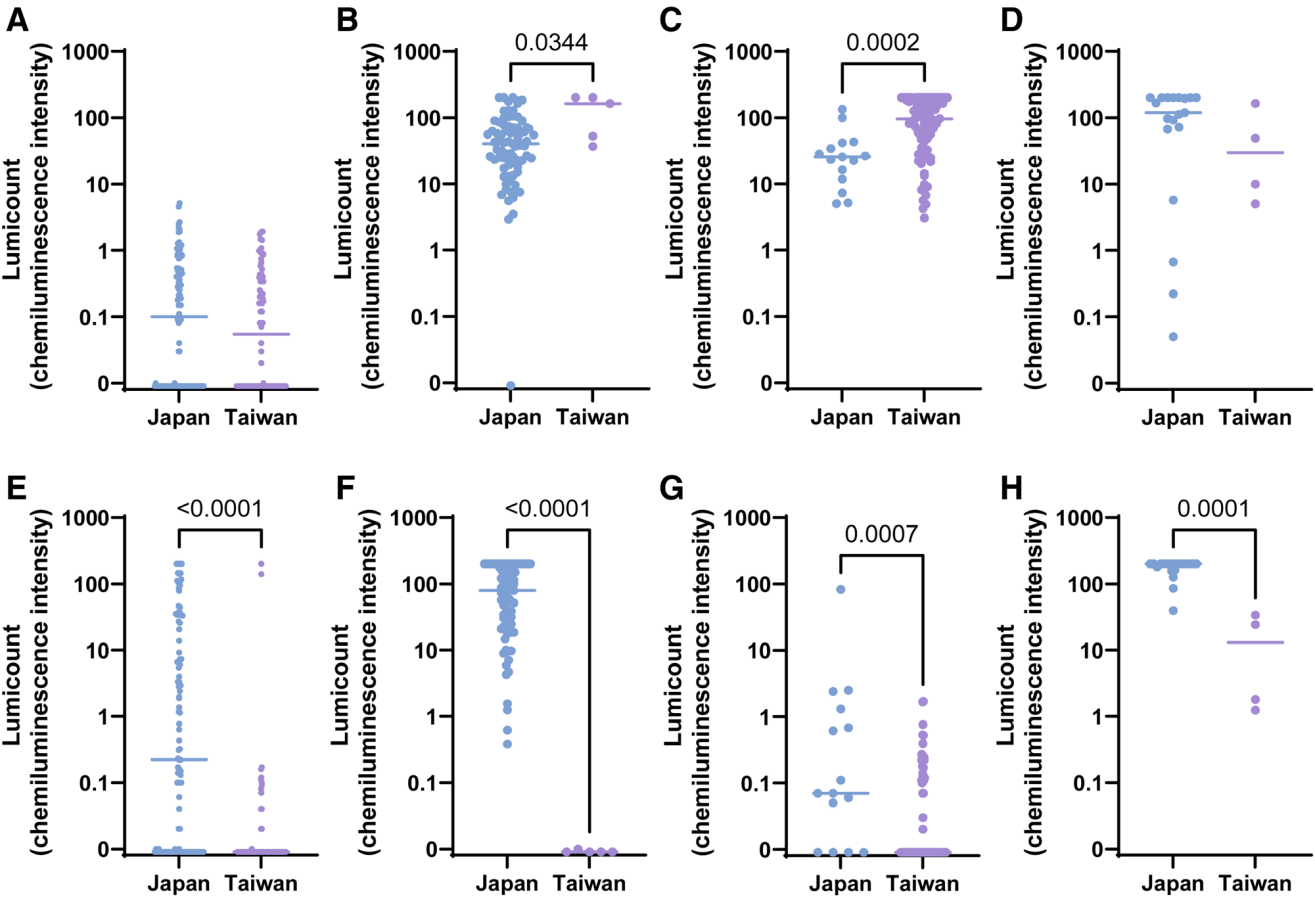
Differences in Lumicount Values for Dermatophagoides farinae (A–D) and Japanese cedar pollen (E–H) across LCA classes in Japan and Taiwan. (A, E) Class 1; (B, F) class 2; (C, G) class 3; and (D, H) class 4. Each panel compares the lumicount values by class and country to highlight class-specific sensitization intensity.

First, for *D. farinae*, the most frequently sensitized allergen, lumicount values were significantly higher in Taiwan than in Japan in classes 2 and 3. As expected, for Japanese cedar pollen, which is a major allergen in Japan, lumicount values were significantly higher in all classes in the Japanese cohort compared with the Taiwanese cohort. Notably, specific IgE to Japanese cedar was also detected at measurable levels in classes 1, 3, and 4 in the Taiwanese cohort, despite the absence of Japanese cedar trees in Taiwan. This may be attributable to cross-reactivity with other structurally related allergens (Fig. 4).

**Figure 5.**
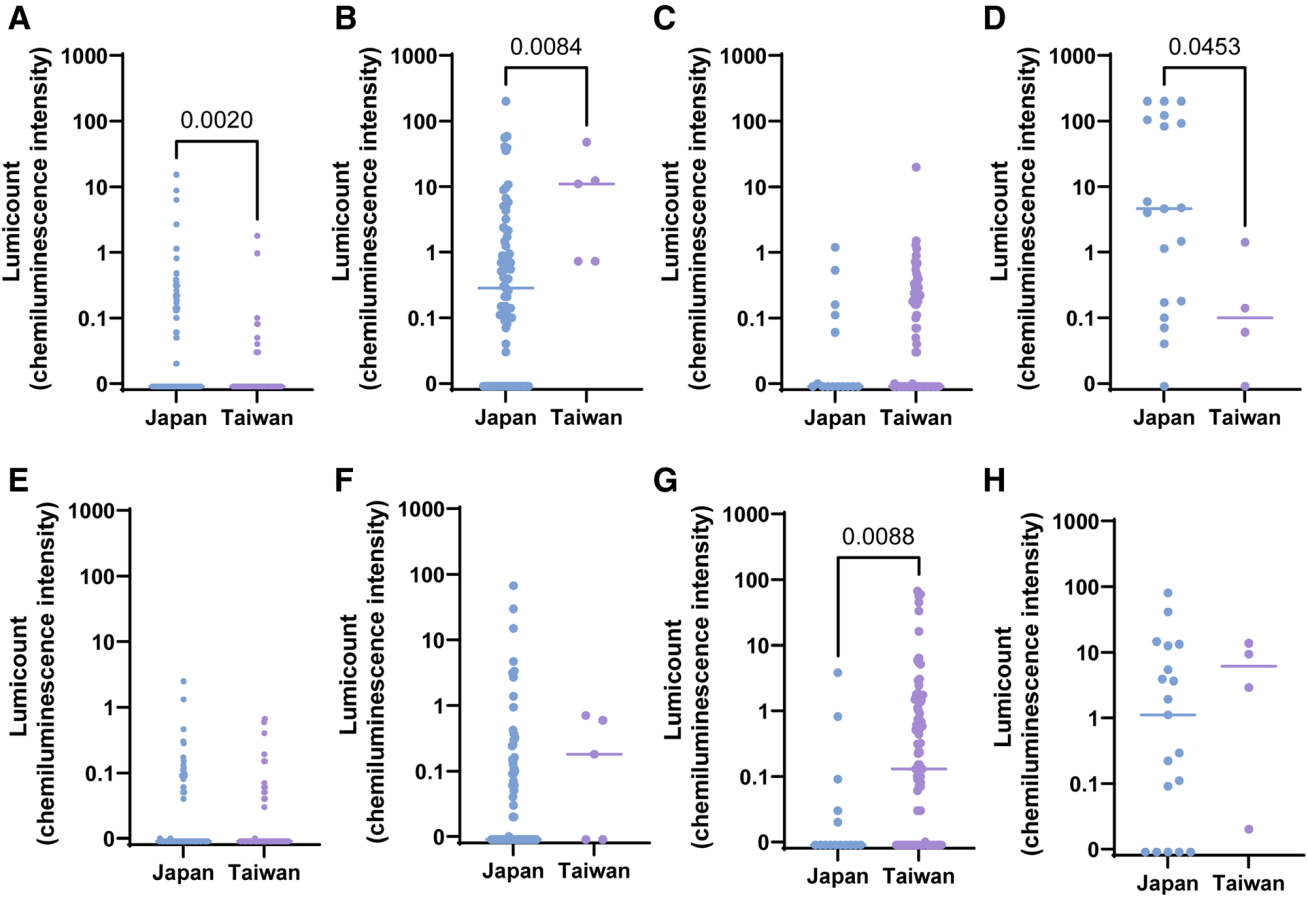
Differences in Lumicount values for dog dander (A–D) and crab (E–H) across LCA classes in Japan and Taiwan. (A, E) Class 1; (B, F) class 2; (C, G) class 3; and (D, H) class 4. The comparison demonstrates the variation in cross-reactive sensitization patterns between the 2 populations.

For dog dander, lumicount values were significantly higher in the Japanese cohort than in the Taiwanese cohort in classes 1, 2, and 4. For crab, significantly higher values were observed in Taiwan compared with Japan in class 3 (Fig. 5).

### Differences in cross-reactivity to HDM and crab between Japan and Taiwan

Cross-reactivity between HDM and crab allergens is well recognized. To assess the strength of this relationship in different populations, we compared the Spearman correlation coefficients between HDM and crab-specific IgE levels in Japanese and Taiwanese children.

The correlation was stronger in the Taiwanese cohort (Spearman’s ρ = 0.5713, 95% confidence interval [CI]: 0.4553–0.6682, n = 166) than in the Japanese cohort (ρ = 0.3937, 95% CI: 0.2713–0.5036, n = 217).

Statistical comparison using Fisher’s z-transformation revealed that this difference was significant (z = 2.24, *P* = 0.025), indicating a significantly stronger correlation between HDM and crab sensitization in the Taiwanese cohort (Supplementary Analysis, https://links.lww.com/PA9/A72).

## Discussion

This study directly compared pediatric allergen sensitization patterns between Japan and Taiwan using a standardized MAST panel combined with LCA. We identified 4 distinct latent classes across both populations, revealing key regional differences. The HDM-dominant class was more prevalent in Taiwan (50%) than in Japan (6.9%), while the IH-S and PS classes were more frequently observed in Japan. The HDM-dominant class was consistently associated with AR and asthma in Taiwan. In contrast, IH-S class, which include HDM sensitization, was associated with AR and asthma in Japan. Additionally, analysis of lumicount values for individual allergens revealed significant between-country differences, such as higher Japanese cedar-specific IgE levels in Japan and higher D. farinae and crab-specific responses in Taiwan.

The higher prevalence of the HDM-dominant class in Taiwan reflects regional environmental conditions, as evidenced by a retrospective analysis from Central Taiwan showing sensitization rates of 41.8% for *D. farinae* and 37.9% for *D. pteronyssinus* [17]. Similar sensitization rates (approximately 40–41%) have been documented in Japan [18], confirming the universal role of dust mites as major allergens. However, despite comparable prevalence, lumicount values for HDM-specific IgE were significantly higher in the Taiwanese cohort, suggesting a greater immunologic burden associated with HDM exposure. This heightened response may be attributed to Taiwan’s subtropical climate, characterized by high humidity, as well as common residential conditions such as limited ventilation and carpeting, which are conducive to dust mite proliferation and allergen accumulation.

Another intriguing finding from this study is that lumicount values for crab-specific IgE were also higher in the Taiwanese cohort. It is well established that HDMs and crustaceans such as crab share a common allergenic protein, tropomyosin, which can lead to cross-reactivity [19]. Interestingly, the correlation between dust mite–specific and crab-specific IgE was significantly stronger in Taiwan, suggesting that the higher dust mite sensitization burden in this population may contribute to secondary sensitization to crab [20]. Although none of the participants in this study reported food allergies, this association may warrant consideration as a potential risk factor for future clinical crab allergy in Taiwanese children. In contrast, the weaker correlation between dust mite and crab sensitization observed in the Japanese cohort may indicate that crab sensitization occurs via alternative, non–dust mite–related pathways. Possible regional or environmental exposure differences—such as dietary habits, indoor allergen profiles, or other environmental factors—could also be considered as contributing to the observed intercohort differences.

In Japan, Japanese cedar sensitization was found to be notably prevalent. This issue was not limited to the IH-S class; even in the LS class, approximately 20% of children exhibited sensitization to Japanese cedar, underscoring its significant impact as a public health concern. Interestingly, a certain level of sensitization to Japanese cedar was also observed in Taiwanese children, despite the absence of this tree species in Taiwan. Rather than suggesting long-distance pollen transport across the sea or isolated instances of Japanese cedar cultivation, this finding is more likely attributable to cross-reactivity with other pollen allergens. Although not shown in the data, we observed a positive correlation between Japanese cedar and Bermuda grass sensitization in the Taiwanese cohort only, based on the Taiwanese allergen panel. While no specific Bermuda grass allergen component has been identified to cross-react with Japanese cedar, this warrants further investigation in future studies.

The multiple sensitization class, more prevalent in Japanese children (8% compared to <4% in Taiwan), appears to capture a broader reactivity pattern, including allergens from seasonal pollens (eg, Japanese cedar and timothy grass) and food proteins. Research has indicated that Pollen Food Allergy Syndrome is relatively common among Japanese children, with significant cross-reactivity between cedar pollen and specific food allergens such as peanuts [21, 22]. These data suggest that in Japan, a diverse environmental allergen exposure, particularly to seasonal pollens, contributes to the risks for multiple sensitizations.

Furthermore, our analysis revealed differences in the distribution of comorbid allergic diseases. In our cohort, the prevalence of AD was higher in Japan, whereas the prevalence of asthma was more pronounced in Taiwan. National data underline these variations: in Taiwan, AD prevalence in children is reported at 9.6% and asthma at 15.7% among those under 20 years of age [23, 24], while in Japan, although overall prevalence rates are comparable (around 14.7% for asthma and 15.6% for AD [25]), regional differences exist (eg, lower AD prevalence in Okinawa) [26].

Genetic susceptibility factors identified in different populations [27, 28] may partly explain the observed disparities, suggesting that the prevalence of allergic diseases within each latent class could be influenced by the distinct genetic backgrounds of Taiwanese and Japanese individuals. In Taiwan, Lu et al. [29] reported 2 novel genetic variants associated with allergic sensitization: an intronic single nucleotide polymorphism (SNP) in the CD28 gene (rs1181388) and an intergenic SNP at chromosome 11q23.2 (rs1002957030). Notably, 7 loci, including these variants, were subsequently replicated in a Japanese meta-analysis. Among human leukocyte antigen (HLA) regions, the HLA-DQA103:02–DQB103:03 haplotype showed the strongest association (odds ratio = 1.25, standard error = 0.02, false discovery rate = 1.6 × 10⁻⁸). Additionally, a genome-wide significant association between the HLA class II region and profilin sensitization was observed (*P* < 5.0 × 10⁻⁸) [29]. These findings collectively support the notion that population-specific genetic predispositions may shape latent sensitization profiles, potentially contributing to the higher prevalence of the HDM-plus-milk phenotype in Taiwan and the prominence of multiple sensitizations in Japan.

A major strength of this study is its novel cross-national design, which integrates the multiplex capacity of MAST with LCA to identify distinct allergen sensitization patterns using a standardized panel of 20 common allergens. This methodological consistency allows for direct comparison between Japan and Taiwan and yields valuable insights into region-specific allergic profiles.

Several limitations should be acknowledged. First, the modest sample sizes, drawn from single tertiary centers in each country, may introduce sampling bias and limit generalizability. Second, the cross-sectional nature of the study precludes analysis of temporal trends and causal relationships. Third, the use of whole allergen extracts, rather than component-resolved diagnostics, may limit specificity in detecting cross-reactivities, particularly among food allergens.

Future studies should adopt larger, multicenter, and longitudinal designs incorporating component-resolved diagnostics alongside genetic and epigenetic analyses. Such approaches would help elucidate the mechanisms underlying regional differences and support the development of personalized allergy management strategies.

## Conclusion

This study revealed distinct allergen sensitization profiles in Japanese and Taiwanese children using MAST and LCA. The findings support the need for region-specific diagnostic approaches and offer a foundation for future research into the mechanisms driving these differences.

## Acknowledgments

The authors sincerely thank the research teams and clinical staff in both Taiwan and Japan for their assistance in data collection and coordination. This study was supported by following grants: NSTC 113-2314-B-039-057 from the National Science and Technology Council, Taiwan; a research grant (1JA8) from the Center for Allergy, Immunology, and Microbiome (A.I.M.), China Medical University Hospital, Taichung, Taiwan; DMR-114-024, and DMR-114-109 from the China Medical University Hospital, Taichung, Taiwan.

## Conflicts of interest

The authors have no financial conflicts of interest.

## Author contributions

Ching-Wei Lin, Su-Boon Yong, and Rei Kanai contributed equally to data collection, analysis, and the initial drafting of the manuscript. Xiao-Ling Liu was responsible for project administration. Mizuho Nagao, Yasunori Sato, and Lawrence Shih-Hsin Wu contributed to data interpretation and critical review of the manuscript. Pei-Chi Chen and Hui-Fang Kao provided technical consultation and methodological support. Chang-Ching Wei, Takao Fujisawa, and Jiu-Yao Wang supervised the study, revised the final manuscript, and provided overall project guidance and funding support. All authors read and approved the final manuscript.

## Data availability statement

The datasets analyzed in this study are not publicly available due to privacy restrictions but are available from the corresponding author upon reasonable request.

## Consent for publication

All authors have read and approved the final version of the manuscript and consent to its submission for publication.

## Ethics statement

This study was approved by the Institutional Review Board of National Cheng Kung University Hospital, Taiwan (IRB No. A-ER-106-105; Chairperson: Thy-Sheng Lin, MD), and by the Ethics Committee of Mie National Hospital, Japan (IRB No. 27-25; Chairperson: Shigeru Suga, MD, PhD). Written informed consent was obtained from the participants’ legal guardians in accordance with local regulations and institutional requirements in both countries.

## Supplementary material

Supplementary Analysis can be found via 10.5415/apallergy.2022.12.e38

Supplementary Analysis

Click here to view

## Supplementary Material
