# Supplementary material for: Distinct regional allergen sensitization patterns in pediatric populations: A comparative latent class analysis of multiple antigen simultaneous test-derived sensitization profiles in Japan and Taiwan
Source: Asia Pac Allergy. 2025 Dec 2;15(4):247–54. doi: 10.5415/apallergy.0000000000000223 (PMC12672179; doi:10.5415/apallergy.0000000000000223)
Supplement: Supplementary file 1 [file pa9-15-247-s001.pdf]

Supplementary Analysis: Correlation between HDM- and Crab-Specific IgE Levels

To evaluate potential cross-reactivity between different allergens, we calculated Spearman's rank correlation coefficients between specific IgE levels, measured as lumicount values. This non-parametric method was used to assess monotonic relationships across allergen-specific IgE responses.

As expected, strong correlations were observed between allergens derived from the same source (e.g., house dust mite and Dermatophagoides farinae). However, we also identified a relatively strong correlation between HDM- and crab-specific IgE levels, suggesting potential cross-reactivity between taxonomically distinct allergens.

To further investigate this observation, we compared the Spearman correlation coefficients between HDM and crab-specific IgE levels in Japanese and Taiwanese cohorts separately.

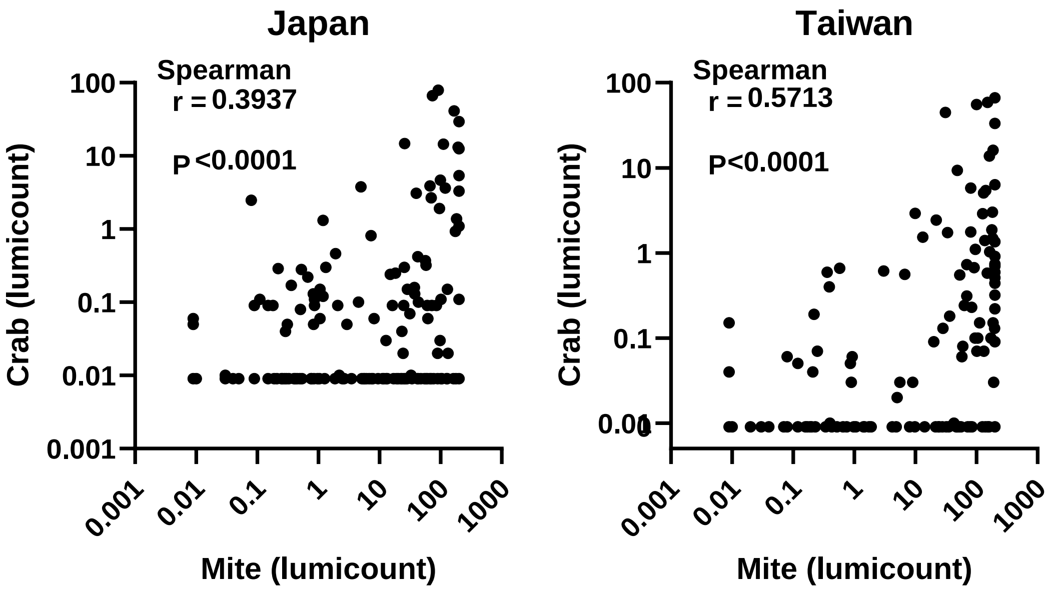

As shown in the figure above, significant correlations were observed in both cohorts. To statistically assess the difference in the strength of these correlations, Fisher's r-to-z transformation was applied. This method approximates the sampling distribution of correlation coefficients as normally distributed and allows hypothesis testing between two independent groups. The z-statistic was calculated based on the transformed values

and respective sample sizes. A two-tailed p-value < 0.05 was considered statistically significant.

The Spearman correlation coefficient in the Taiwanese cohort (n = 166) was 0.5713 (95% CI: 0.4553–0.6682), whereas in the Japanese cohort (n = 217), it was 0.3937 (95% CI: 0.2713–0.5036). Fisher's z-test yielded a z-statistic of 2.24 and a corresponding two-tailed p-value of 0.025, indicating a statistically significant difference in the strength of correlation between the two populations.
